# Temperature-Driven Stopped-Flow Experiments for Investigating the Initial Aggregation of the α-Synuclein Amyloid Protein, Focusing on Active and Inactive Phases

**DOI:** 10.1007/s10895-024-03971-8

**Published:** 2024-10-02

**Authors:** Marco A. Saraiva

**Affiliations:** 1 Centro de Química Estrutural, Institute of Molecular Sciences, Instituto Superior Técnico, University of Lisbon, Campus Alameda, Av. Rovisco Pais, Lisbon, 1049-001 Portugal; 2 Instituto de Tecnologia Química e Biológica António Xavier, Universidade Nova de Lisboa, Av. da República, Oeiras, 2780-157 Portugal

**Keywords:** a-synuclein, Stopped-flow, Temperature-jumps, Steady-state, Burst phase, Protein aggregation and disaggregation

## Abstract

The primary objective of this research is to further examine the events occurring during the active or burst phase by focusing on the aggregation of the Syn amyloid protein. Regarding this aspect, it was initially conducted rapid temperature variations using stopped-flow spectrometry and tyrosyl group fluorescence emission detection, within the initial 500 milliseconds in buffered Syn solutions at pH 7, exploring various temperature ranges to investigate protein aggregation. The results obtained were contrasted with results obtained for the *N*_α_-acetyl-L-tyrosinamide (NAYA) parent compound in the same conditions. The utilization of the NAYA compound is suitable as it mimics the peptide bonds in proteins and contains a tyrosyl group resembling the four tyrosyl groups found in the Syn protein structure (the protein has no tryptophan residues). Furthermore, the NAYA compound adopts an intramolecularly hydrogen-bonded structure even in an aqueous solution, similar to the interactions seen in the hydrophilic face of β-sheets. Additionally, the Syn protein system can exhibit the presence of β-sheets as a result of the existence of very low abundant Syn amyloid precursor forms or nuclei during the initial stages of the protein aggregation. Thus, a relationship is present between the molecular processes in the NAYA and Syn protein systems, making the NAYA’s application crucial in this research. Moreover, to aid in understanding the results, it was also compared the events during the quiescent or inactive phase (30–500 milliseconds) with those in the burst phase (up to 10 milliseconds) using stopped-flow spectrometry conditions. Steady-state measurements were beneficial in comprehending the occurrences in both the quiescent and burst phases examined. Although protein aggregation and disaggregation were observed during the quiescent phase, determining these processes in the burst phase was more challenging. In the latter case, the aggregation of the Syn protein is actually initiated by the interaction of the intrinsically disordered Syn monomers. In the quiescent phase, first-order rate constants were measured and analysis showed that Syn protein aggregation and disaggregation occur simultaneously. At lower temperatures, early protein disaggregation outweighs protein aggregation whereas at higher temperatures protein disaggregation and aggregation are rather similar. It is also need to highlight that the burst phase, while distinct from the quiescent phase, can be considered as a possible structural phase for obtaining details about the aggregation of this specific disordered protein in solution on a very short timescale.

**Supplementary Information:**

The online version contains supplementary material available at 10.1007/s10895-024-03971-8.

## Introduction

A method that has gained considerable interest refers to creating closed systems to research non-equilibrium supramolecular polymerizations of compounds [1]. Despite being mechanosensitive and dissipative, these closed systems exhibit similarities to the system responsible for stopped-flow spectrometry. The latter is also a sealed system that allows for the examination of solutions in conditions where there is turbulence, such as the active or burst phase [2]. However, the occurrences pertaining to the burst phase include micromixing impacts that are non-mechanosensitive dissipative. One of the goals of this research is to uncover new interactions of matter in a short timescale by observing the events during the burst phase, where non-equilibrium conditions emerge. The burst phase events occur in the initial 20 milliseconds [2]. In a prior study, we explored the burst phase behavior of the disordered amyloid α-synuclein (Syn) protein, finding that a folding intermediate appears at pH 3 but not at neutral pH [2]. Raising the temperature is recognized to boost the aggregation of the Syn protein [3]. We examined both the inactive or quiescent phase (ranging from 30 to 500 milliseconds) and the burst phase (lasting up to 10 milliseconds). It is crucial to highlight that during the burst phase, the Syn protein, which is natively unfolded, remains in a non-collapsed state at pH 7 [2]. Hence, it is highly probable that the amyloid protein in its unfolded state at pH 7 may transition to an aggregated state, similar to what was seen with different protein concentrations [2]. It’s crucial to note that, during the burst phase, the natively unfolded Syn protein may transition to an aggregated state, while in the quiescent phase, small amounts of protein aggregates, very likely Syn amyloid precursor forms, are present at an early stage, as mentioned later on.

In 2020, we initially noted the development of early sizable Syn aggregates, possibly Syn amyloid precursor forms, in the protein solutions within less than 1 h through DLS (with a hydrodynamic radius exceeding 100 nm) [4]. The Syn protein stock solutions were centrifuged using 100 kDa membrane centrifuge filters, thus large protein aggregates are detected early in the solution, not from potential impurities in the purification process [4]. It should be emphasized that early-stage large aggregates of Syn are responsive to changes in pH and ionic strength in protein solutions [4–6]. According to the number distributions measured by DLS, we also found that the initial protein aggregates have a concentration of approximately 10^− 9^ – 10^− 10^ M [4 − 6]. This means that there is a concentration difference of four orders of magnitude between the Syn monomers and the initial large Syn aggregates [4 − 6]. Additionally, another group has recently stated that Syn aggregates (with a hydrodynamic radius exceeding 100 nm) were formed during the lag-phase, which have been shown to be toxic to cells [7]. Aggregates have also been noticed for the Aβ42 amyloid peptide during the lag phase [8]. It has been very recently reported that oligomeric species formed predominantly on aggregates (fibrils) surfaces, a broad class which includes the bulk of oligomers formed by the key Alzheimer’s disease-associated Aβ peptides, also dissociate overwhelmingly on aggregates’ surfaces, not in solution as had previously been assumed [9, 10]. Therefore, it can be anticipated that early Syn aggregates (fibrils) play a role in facilitating the formation of smaller amyloid species. It is further to mention that spontaneous formation of amyloid fibrils from disordered peptides is often discussed as a version of a nucleated growth polymerization pathway. In this mechanism, the overall rate of amyloid formation is limited by the slow generation of a nucleus (the nucleation phase), which, once formed, rapidly grow by monomer addition to the fibril ends (the elongation phase) [11]. However, in many amyloid systems, this mechanism is complicated by the early formation of a variety of noncovalent oligomeric structures [12], but simple polyglutamine peptides do not appear to form such oligomers under native conditions, allowing relatively straightforward study of both kinetic phases of amyloid growth [11, 13]. Furthermore, the aggregation kinetics data show that the N-terminal hepta-peptide sequence of yeast prion protein *Sup*35 with the sequence GNNQQNY follows nucleation-dependent aggregation kinetics with a critical nucleus of size ∼ 7 monomers and that the efficiency of nucleation were found to be inversely related to the reaction temperature [14]. The nucleus reduces the thermodynamic energy barrier by acting as a template for further self-assembly and results in highly ordered amyloid fibrils [14].

Additionally, Syn protein aggregation is linked to the onset of Parkinson’s disease, with brain inclusions known as Lewy bodies containing aggregated Syn [15, 16]. Research on Syn protein aggregation has been extensive in the past twenty years, yet the exact process of its aggregation remains partially unclear. Investigating early Syn aggregates, also known as Syn nucleus and oligomers, is difficult because they are heterogeneous, transient, and exist in very small quantities [17, 18]. Late stage species, known as mature Syn fibrils, are the most stable and definitely the most extensively researched [19–21]. The monomeric Syn protein consists of 140 amino acid residues (14.5 kDa) and it is composed by three distinct regions in its molecular structure. The NAC region, located in the central region (61–95), is responsible for causing protein fibrillation. The N-terminal region (1–61) shows some tendency towards helical structure and can be further categorized into H1 (1–31) and H2 (32–61), whereas the extremely acidic C-terminal region (96–140) is disordered.

In this study, it was compared stopped-flow spectrometry and steady-state measurements to find that intermolecular events were observed during both the quiescent phase and the burst phase for the Syn amyloid protein. In the burst phase, under turbulent and micromixing conditions as similarly to agitation conditions [22], the Syn aggregation is enhanced. Due to development of the gas-liquid interface during micromixing and cavitation formation the primary reacting species appear to be the Syn protein monomers. In the quiescent phase, the Syn aggregation reaction proceeds at a much slower rate than that with agitation [22] and in such stagnant conditions the interactions of Syn amyloid precursor forms at amyloid fibrils ends are more deficient leading to a decreased elongation phase. The above features are actually predicted by the variations of the recorded Syn tyrosyl groups fluorescence intensity in both the burst and quiescent phases examined. Moreover, through comparing the occurrences in the quiescent phase to those in the burst phase, it was determined that these phases can provide additional information when combined. Moreover, this research emphasizes the opportunity to explore the burst phase as a potential structural phase, especially in relation to understanding the aggregation of amyloid proteins in a short timescale.

## Materials and Methods

### Materials

The *N*_α_-acetyl-L-tyrosinamide hydrochloride (NAYA) compound was purchased from Sigma-Aldrich.

### Syn Expression and Purification

The pT7-7 plasmid containing the human Syn sequence (kindly provided by Professor Doctor T. Outeiro, IMM, University of Lisbon) was used to overexpress Syn in *Escherichia coli* BL21(DE3) bacteria. Syn was purified as previously described [2, 4–6, 23–27].

### Dynamic Light Scattering (DLS) Measurements

Particle diffusion coefficients were measured by DLS using a Zetasizer Nano ZS device (He–Ne red laser (633 nm), Malvern Instruments) as previously described [4–6]. It should be noted that the particle diffusion coefficients were derived from analyzing the obtained intensity autocorrelation function. In addition, the Malvern general purpose algorithm was used, which indicates an α parameter or “regularizer” of 0.01. Accordingly, the indicated α parameter value is best suited for the analysis of protein samples using Malvern instruments, as tested for lysozyme (0.3 mg/mL in PBS buffer at pH 6.8) and denatured hemoglobin at 44 ºC (PBS buffer at pH 6.8). The Syn protein concentration of 33.5 µM (*A*_275 nm_ = 0.2; 0.5 mg/mL) at a solution pH of 7 was optimized for DLS experiments to ensure that instrument count rates were in the range of 200–500 kcps (kilo counts per second) [4–6]. All the material used was carefully cleaned and manipulated to avoid dust particles, and the solutions of buffer and diluted HCl were previously filtered with 0.22 µm pore filters. The Syn stock solutions used in the DLS measurements were filtered by centrifugation with a 100 kDa MWCO (molecular weight cut-off) membrane centrifuge filter (Amicon, the membrane was made of regenerated cellulose) (centrifugation conditions: 5,000 rpm (3,214*g*, relative centrifugal force (RCF)) at 4 ºC for 3 minutes (1 mL protein stock sample), fixed-angle rotor, Eppendorf Centrifuge 5810). A simple protocol based on filtration through a 100 kDa MWCO membrane has been reported to provide an efficient method for producing an aggregate-free Syn preparation and for rapid removal or isolation of Syn oligomers (> Syn dimers) [28]. Sample preparation: 1 mL of protein solution in 10 mM tris-HCl with a concentration of 33.5 µM at the desired pH (prepared with high-purity Milli-Q water) was carefully transferred to a quartz cuvette (Hellma Analytics – QS 10.00 mm), and experiments were performed at 20.0 ºC. The number of DLS runs was automatic. The duration of each run was approximately 8 seconds. In addition, the number of runs was similar for each DLS measurement. The samples were equilibrated at 20.0°C for 120 seconds and the data were recorded continuously.

### Steady-State Fluorescence Intensity Measurements

Steady-state fluorescence emission spectra were measured using a SPEX Fluorolog 212I spectrofluorimeter. Fluorescence emission spectra were collected in the S/R mode and in the right angle geometry. In fluorescence, 5 mm path length quartz fused cuvettes were used. The NAYA compound concentration of 2.9 × 10^− 4^ M (*A*_275 nm_ = 0.4; ε = 1390 M^− 1^ cm^− 1^) (10 mM tris-HCl, pH 7) and the Syn protein concentration of 67.0 µM (*A*_275 nm_ = 0.4; ε = 5974 M^− 1^ cm^− 1^) (10 mM tris-HCl, pH 7) were used in the determination of the fluorescence emission spectra. The fluorescence quantum yield of Syn (0.029 at 25 °C) was determined by comparison with the quantum yield of NAYA in water (0.047 at 25 °C) [23].

### Time-Correlated Single Photon Counting (TCSPC) Measurements

Fluorescence decays were measured using the time-correlated single photon counting technique as previously described [23]. The excitation was provided by the frequency-tripled emission of a Millennia Xs/Tsunami lasers system from Spectra Physics, operating at 82 MHz. The fluorescence emission was collected at the magic angle (Glan-Thompson polarizer), passed through a monochromator (Jobin-Yvon H20 Vis) and detected with a microchannel plate photomultiplier (Hamamatsu R3809u-50). The full width at half-maximum (FWHM) of the instrumental response was ca. 18 ps with 814 fs/channel resolution. The decays were generally measured with 5000 counts at the peak and deconvoluted from the IRF using the modulations function method (Sand program) [23].

### SFM-4 Stopped-Flow Fast-Mixing Temperature-Jump Measurements

Fast-mixing temperature-jump measurements were carried out using a temperature jump accessory from BioLogic coupled to an existing BioLogic SFM-4 with an MPS-52 microprocessor unit connected via a fiber optic cable to a xenon-mercury lamp equipped with a circulating water bath for temperature control [29]. The accessory achieves temperature jumps as high as 40 °C by rapid mixing of two solutions initially at different temperatures, *T*_1_ and *T*_2_. The final temperature of the mixture (*T*_f_) is determined both by the initial temperatures *T*_1_ and *T*_2_ and by the volume ratio of the two solutions. Three Peltier elements (BioLogic TCU-250) are used to control the initial temperatures of the two solutions and the observation cell after mixing. The heat generated by the Peltier elements is dissipated using a water bath circulator. Prior to the temperature-jump experiments for each *T*_f_, test shots were carried out for both heating and cooling to determine the optimal conditions of (1) the appropriate voltage (*V*) for a sufficiently strong signal intensity without saturation and (2) the correct flow rates (*Q*) to ensure that the temperature is kept constant in the region between the mixer chamber and the observation cell (*Q*_f_ = 12 mL/s) [29]. For each *T*_f_, the following data were collected: temperature-jumps for heating (5 °C to *T*_f_ and 25 ºC to *T*_f_) and cooling (30 °C to *T*_f_ and 70 °C to *T*_f_), and shots with only NAYA, Syn and buffer both at the same *T*_f_, to guarantee that the three Peltier elements are intercalibrated and to ensure that upon mixing of protein and buffer no deviations are observed from the temperature (*T*_f_) of the observation cell, which, if present, could lead to artefacts in kinetic curves. The fast-mixing temperature-jumps were performed by recording the changes in the NAYA compound and the Syn protein intrinsic fluorescence of its tyrosyl groups with excitation at *λ* = 275 nm, and the emission was collected above 290 nm using a 290 nm Schott cut-off filter [2, 26]. The NAYA compound concentration of 2.9 × 10^− 4^ M (*A*_275 nm_ = 0.4; ε = 1390 M^− 1^ cm^− 1^) (10 mM tris-HCl, pH 7) and the Syn protein concentration of 67.0 µM (*A*_275 nm_ = 0.4; ε = 5974 M^− 1^ cm^− 1^) (10 mM tris-HCl, pH 7) were used in the performed fast-mixing temperature-jumps. It is to be noted that the Syn centrifuged protein solution is aged for approximately 30 min (time necessary for protein concentration determination and for preparing the stopped-flow device) before the heating and cooling measurements by fast-mixing temperature-jumps.

## Results and Discussion

In this report, it was determined that Syn amyloid precursor forms with very low abundance are present early in the protein solutions (refer to *Supplementary Materials* for more information). It should be noted that although elongated forms of amyloid precursor were expected in the Syn protein solutions at neutral pH based on initial DLS autocorrelation function simulation (*Supplementary Materials*), additional research is needed to fully characterize these amyloid precursor species. Because there are limited amounts of the elongated amyloid precursor forms identified by DLS, technical limitations arise with utilizing existing methodologies.

**Scheme 1:**
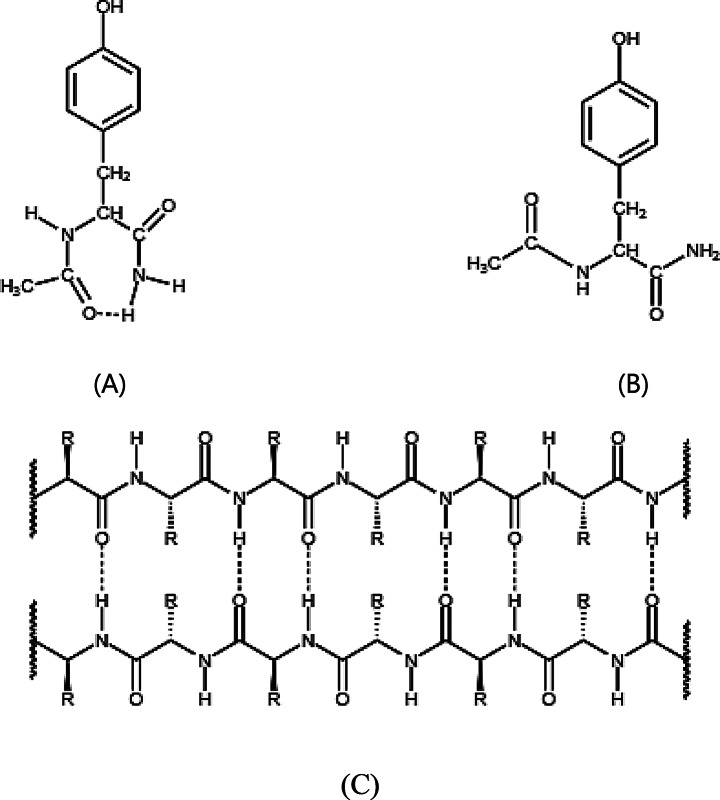
Relation between NAYA and protein β-sheet hydrogen-bonded interactions. (**A**) NAYA closed shape configuration, including the amide intramolecular hydrogen-bonded interaction. (**B**) NAYA extended configuration. (**C**) Protein β-sheet structure showing, in the hydrophilic face (β-sheets possess hydrophilic and hydrophobic faces), several hydrogen-bonded interactions, being these latter similar to the NAYA amide intramolecular hydrogen-bonded interaction displayed in (**A**)

In 1972, J. R. Cann reported that acetylamino acid methylamides can form an intramolecularly hydrogen-bonded configuration (Scheme 1A) in nonpolar solvents such as CCl_4_ and 1,4-dioxane [30]. The NAYA compound is among the compounds included [30]. The creation of this seven-membered ring with an intramolecular hydrogen bond (Scheme 1A) is likely preferred in nonpolar solvents and lower temperatures [30–32]. It is anticipated that in pure water, this NAYA compound species will only be present in very small quantities due to the nature of water as a solvent capable of forming hydrogen bonds (donor or acceptor) and thus promoting the disruption of the complex. Previous studies have shown tyrosyl group fluorescence intensity decays of the NAYA compound in both water and trifluoroethanol, by being double exponential, provide additional support for the presence of the intramolecularly hydrogen-bonded NAYA species [33]. This type of solvents is able to donate hydrogen bonds, which may not easily disrupt the existing NAYA intramolecular hydrogen bond. Additionally, the shorter decay time of the NAYA parent compound persists up to 50 ºC in pure water during its tyrosyl group fluorescence decay process [33]. Collectively, these findings suggest that the NAYA parent compound is most likely to form a stable intramolecularly hydrogen-bonded structure in the presence of hydrogen-bond donor solvents and at low temperatures. This argument suggests that a balance may exist between the NAYA compound’s intramolecularly hydrogen-bonded configuration (Scheme 1A) and the disrupted complex (extended NAYA configuration (Scheme 1B)) when increasing the temperature of NAYA compound in pure water. The involvement of the NAYA compound is crucial in this research as the NAYA compound have an intramolecular hydrogen-bonded structure (Scheme 1A) that resembles the interactions found in the hydrophilic face of β-sheets (Scheme 1C). The latter structures can be present in Syn amyloid precursor forms [11].

### Temperature Measurements for the Syn Protein Under Steady-State Conditions

**Fig. 1:**
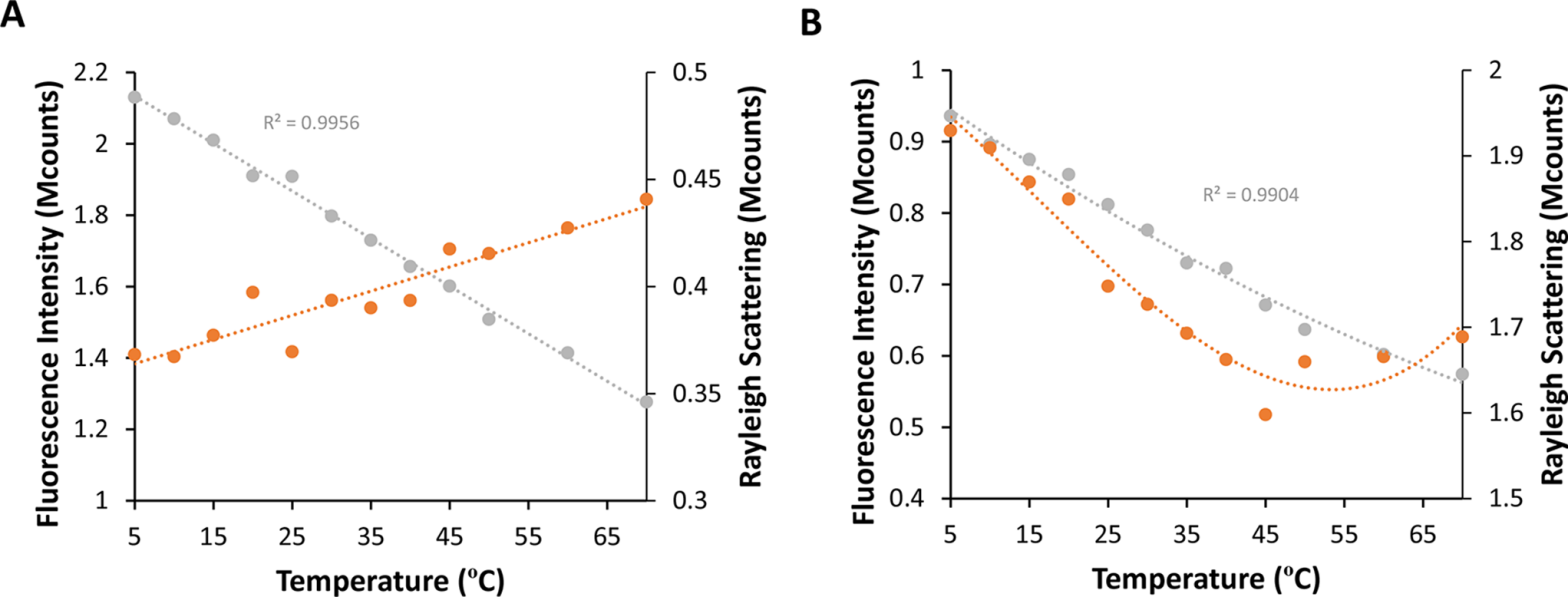
NAYA parent compound and Syn amyloid protein under steady-state conditions while varying the temperature of the solution (10 mM tris-HCl, pH 7). (**A**) Recorded fluorescence intensity (grey data points) and Rayleigh scattering (orange data points) for the NAYA parent compound (*A*_275 nm_ = 0.4; 1390 M^− 1^ cm^− 1^; 2.9 × 10^− 4^ M). (**B**) Recorded intrinsic fluorescence intensity (grey data points) and Rayleigh scattering (orange data points) for the Syn protein (*A*_275 nm_ = 0.4; 5974 M^− 1^ cm^− 1^; 67.0 µM; 1 mg/mL). Every solution was kept at the specified temperature for 5 min to guarantee stability before recording the spectroscopic signals

Prior to showcasing the results from the stopped-flow experiments, it was initially displayed the findings gathered from steady-state conditions for the NAYA parent compound and the Syn amyloid protein. Figure 1A displays both the recorded fluorescence intensity and Rayleigh scattering for the NAYA parent compound under steady-state conditions. It can be clearly seen in this figure that the fluorescence intensity decreases linearly as the temperature rises for the NAYA parent compound, which is an expected finding. Therefore, the rise in temperature causes a rise in solution entropy and favors solute and solvent (water) molecules collisions, leading to a steady decrease in the recorded fluorescence intensity of the NAYA tyrosyl group. Furthermore, Fig. 1A depicts a gradual rise in Rayleigh scattering observed for NAYA as the temperature increases. This suggests a small NAYA compound self-interaction when the temperature increases. It is possible a priori because as the temperature rises in NAYA aqueous solutions, there is a higher electrostatic repulsion in the medium, leading to a decrease in the dielectric constant of the medium, favoring in turn the establishment of hydrophobic interactions. These conditions make it easier for the NAYA parent compound molecules to interact with themselves. Although solvent effects are present, it is expected that raising the temperature of NAYA aqueous solutions will lead to the destabilization of the intramolecularly hydrogen-bonded structure of the NAYA compound, as shown in Scheme 1A. As a result, the formation of non-complexed or extended NAYA compound structure, as depicted in Scheme 1B, is favored. Figure 1B displays the impact of increasing the temperature of Syn solutions on both the measured Syn intrinsic fluorescence intensity and Rayleigh scattering under steady-state conditions. In Fig. 1B, like Fig. 1A, there is a consistent drop in the intrinsic Syn fluorescence intensity as the temperature increases, as expected. An additional aspect is the rise in Syn Rayleigh scattering observed in comparison to the NAYA parent compound, which can be attributed to the increase in Syn protein aggregation. An in-depth analysis of Fig. 1B shows that at lower temperatures such as 5 ºC, the Rayleigh scattering of Syn has higher values, which decrease as the temperature reaches around 45 ºC. After this subsequent rise in temperature, the Rayleigh scattering of the Syn protein experiences a small increase (Fig. 1B). In accordance with Fig. 1, the molecular behavior is rather comparable in both the NAYA and Syn systems as the temperature of the solutions rises. Increasing the temperature disrupts the intramolecular hydrogen bonds in the NAYA compound, indicating NAYA self-association, as stated. Disaggregation of the Syn protein occurs as the temperature rises due to potential disruption of the intramolecular hydrogen-bonded interactions similar to NAYA, leading to the dissociation of current β-sheets in the early-formed Syn amyloid precursor forms. It is to mention that the main type of interaction observed in the hydrophilic face of the early-formed Syn amyloid precursor forms structures (Scheme 1C) is the NAYA-like amide hydrogen-bonding (Scheme 1A). It is important to also note that in the complex form (Scheme 1A), NAYA is in a locked configuration, reducing complexity by eliminating multiple tyrosyl group rotamers populations and complexed interconversion rates, resulting in clear fluorescence emission from the NAYA tyrosyl group under steady-state conditions. This characteristic is actually anticipated for Syn tyrosyl groups located in the hydrophilic face of β-sheets possibly existing in initial Syn amyloid precursor forms. This is why it’s beneficial to use the NAYA complex form as a model for early-formed Syn amyloid precursor forms.

### Time-Correlated Single Photon Counting Data Analysis to Unravel the Contribution of Syn Tyrosyl Groups in the Process of the Protein Aggregation Under Steady-State Conditions

**Fig. 2:**
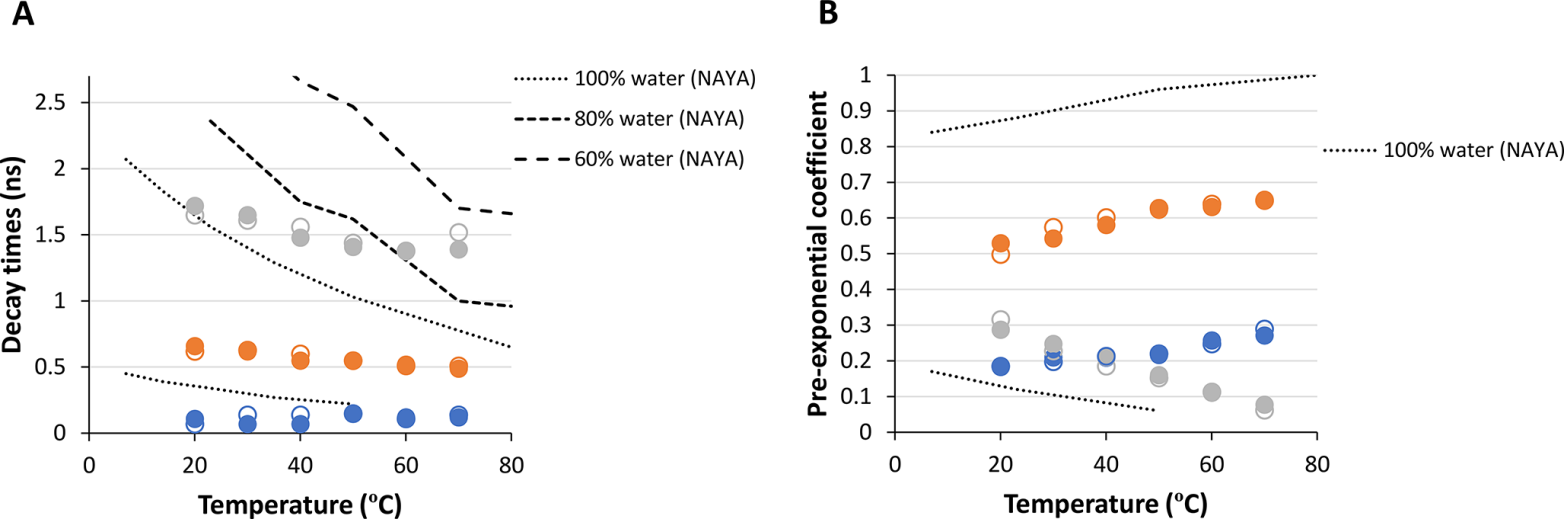
Fluorescence decay times (**A**) and pre-exponential coefficients (**B**) determined for Syn protein solutions with a 67.0 µM protein concentration (10 mM tris-HCl, pH 7) with λ_ex_ = 278 nm and λ_em_ = 295 nm. Heating (closed symbols) and cooling (open symbols) are indicated as well as data for the NAYA compound in water and in the presence of 1,4-dioxane [33]

Here, it was determined the contribution of the four Syn tyrosyl groups in the referred amyloid protein aggregation under steady-state conditions by conducting experiments using time-correlated single photon counting technique (TCSPC). Figure 2A displays the fluorescence lifetimes obtained for the Syn protein and the NAYA parent compound in terms of tyrosyl group fluorescence emission. The dashed lines in Fig. 2A display the NAYA fluorescence lifetimes when in water and in the presence of 1,4-dioxane (a non-polar, water-miscible solvent) [33]. The variation of two fluorescence lifetimes of NAYA in water was noticed by increasing the temperature of NAYA solutions (Fig. 1A). In 10 mM tris-HCl at pH 7, Syn exhibits mostly changes in one of the three fluorescence lifetimes as the temperature of the protein solution rises (Fig. 2A). Regarding the NAYA compound, as discussed earlier, the variation in the longer fluorescence lifetime is attributed to the extended NAYA configuration (Scheme 1B) while the slight variation in shorter fluorescence lifetime is linked to the complexed NAYA configuration (Scheme 1A). In the case of the Syn protein, the longer fluorescence lifetime variation is actually caused by the Syn tyrosyl groups in the possible β-sheets face of Syn amyloid precursor forms, while the slight variations in the two shorter fluorescence lifetimes are linked to the dissociation of the possibly formed β-sheets. This can be attributed to the fact that there is no significant change in fluorescence lifetimes as the solution temperature increases, as long as the tyrosyl groups from the NAYA compound or Syn protein structures are completely exposed to water. Considering the variation of the longer fluorescence lifetime of the Syn protein, it can be implied based on observations with data from the NAYA compound in water and in the presence of 1,4-dioxane that the tyrosyl groups in the protein are able to detect a somewhat hydrophobic environment up to around 80% of the water (Fig. 2A). This when the temperature of the protein solution reaches 60 ºC (Fig. 2A). This suggests that the four Syn tyrosyl groups probably perceive a similar environment, which is almost water. In spite of this, there is a minor rise in the hydrophobicity detected by the Syn tyrosyl groups as the temperature of the protein solution increases, which is anticipated because of the increased aggregation of the protein under these circumstances (Fig. 2A). It should be noted that two shorter fluorescence lifetimes for the Syn protein which remain almost invariable with temperature increase, result in a total of three fluorescence lifetimes for the Syn tyrosyl groups, as mentioned. The tyrosyl group in the NAYA compound shows only two fluorescence lifetimes. This feature has already been explained using the three rotamers model, where the *gauche*(+) rotamer is barely present in the NAYA compound [34]. However, for tri-peptides and proteins, the additional rotamer population mentioned becomes important [34]. Figure 2B shows the change in the pre-exponential coefficients or amplitudes for the fluorescence lifetimes of NAYA compound and Syn protein tyrosyl groups. Amplitudes for the shorter fluorescence lifetimes of the Syn protein rise with increasing temperature of the protein solution, while the amplitude of the shorter fluorescence lifetime of the NAYA compound decreases under the same conditions (Fig. 2B). Hence, one could suggest that the amplitudes related to the shorter fluorescence lifetimes of Syn, in which the latter correspond to the non-complexed state of β-sheets, indicate for the potential dissociation of the formed protein β-sheets. To further strengthen, the amplitudes linked to Syn’s longer fluorescence lifetime decrease as the protein solutions’ temperature rises (Fig. 2B). Increasing the temperature of Syn solutions might lead to the dissociation of early-formed β-sheets.

### Fast-Mixing Temperature-Jumps for the Syn Protein in the Quiescent Phase Under Stopped-Flow Spectrometry Conditions

**Fig. 3:**
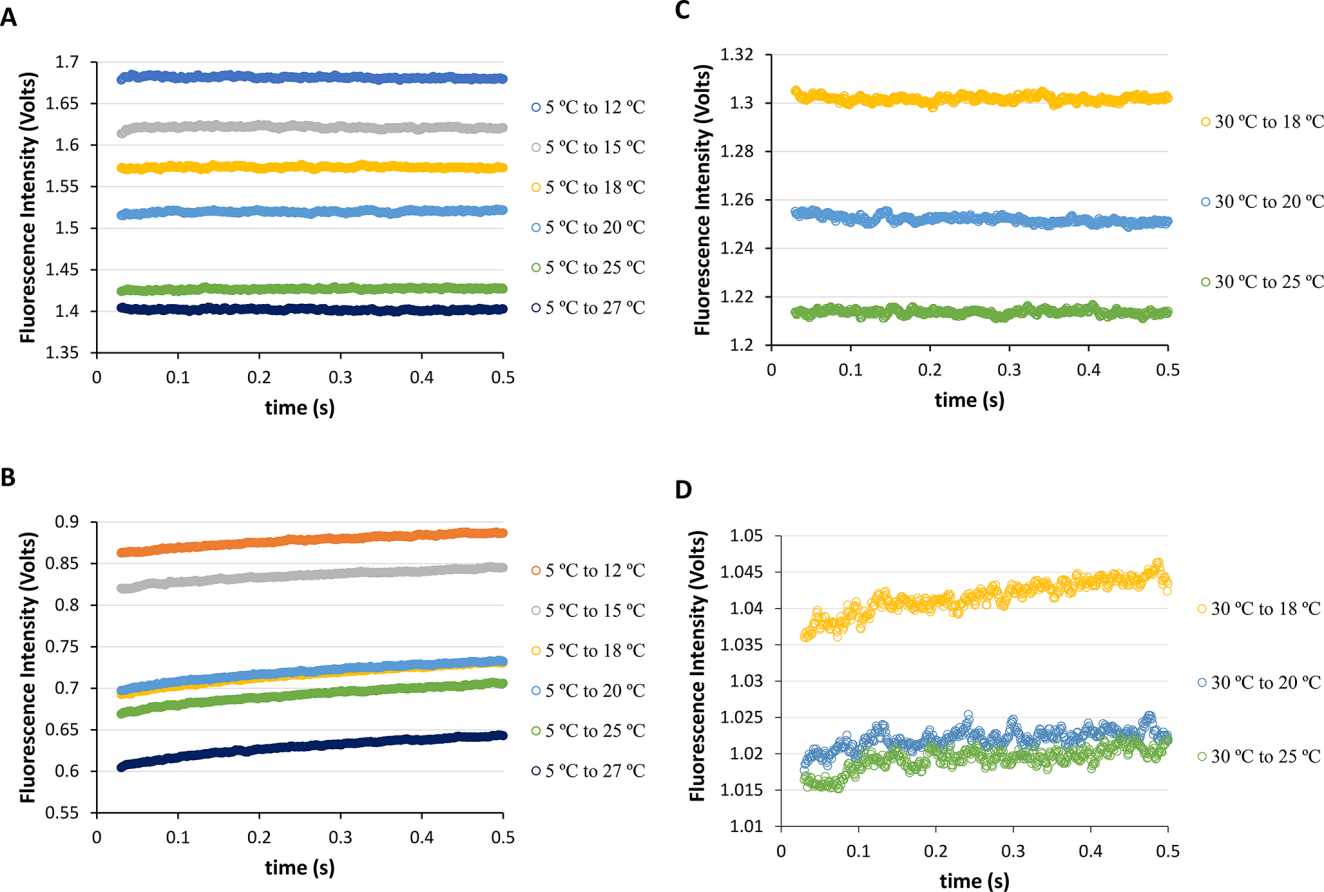
Fast-mixing temperature-jumps for the NAYA parent compound and for the Syn protein (10 mM tris-HCl, pH 7; *A*_275 nm_ = 0.4) in the first 30–500 milliseconds. (**A**) NAYA fluorescence intensity variation in the temperature-jumps from *T*_i_ = 5 ºC to *T*_f_ = 12, 15, 18, 20, 25 and 27 ºC (heating). (**B**) Syn intrinsic fluorescence intensity variation in the temperature-jumps from *T*_i_ = 5 ºC to *T*_f_ = 12, 15, 18, 20, 25 and 27 ºC (heating). (**C**) NAYA fluorescence intensity variation in the temperature-jumps from *T*_i_ = 30 ºC to *T*_f_ = 18, 20 and 25 ºC (cooling). (**D**) Syn intrinsic fluorescence intensity variation in the temperature-jumps from *T*_i_ = 30 ºC to *T*_f_ = 18, 20 and 25 ºC (cooling). The temperature-jumps were performed in triplicate and for clarity only the average of the results is presented

Accordingly, with information from steady-state conditions (Sect. 3.1 and 3.2), we are now in position to reveal the results obtained from executing fast-mixing temperature-jumps for both the NAYA parent compound and the Syn protein using stopped-flow spectrometry. It was chosen a starting temperature of 5 ºC because it is similar to the temperature used in protein chromatographic purification (4 ºC) to investigate the effects on the NAYA parent compound and, in particular, the Syn protein solutions. So, fast-mixing temperature-jumps on buffered NAYA and Syn protein solutions at pH 7 from an initial temperature (*T*_*i*_) of 5 ºC to final temperatures (*T*_*f*_) of 12, 15, 18, 20, 25 and 27 ºC (heating) were conducted (Fig. 3A and B). Specifically, the fluorescence intensity of the NAYA parent compound in Fig. 3A shows no significant change during the initial 500 milliseconds of the heating process. On the contrary, the measured fluorescence intensity for the Syn protein increases in Fig. 3B under identical conditions. Similarly observed in steady-state conditions, the NAYA compound solutions exhibit a greater reduction in fluorescence intensity during the heating process compared to the Syn protein under the same conditions. It is evident from Fig. 3B that the observed intrinsic fluorescence intensity of Syn rises over time, suggesting that Syn aggregation occurs within the initial 500 milliseconds of the fast-mixing temperature-jumps. We also chose to examine the cooling procedure of both the NAYA compound and the Syn protein solutions (Fig. 3C and D). In this context, it was conducted fast-mixing temperature-jumps in buffered NAYA and Syn protein solutions at pH 7, lowering the temperature from *T*_*i*_ = 30 ºC to *T*_*f*_ = 18, 20, and 25 ºC (cooling) (Fig. 3C and D). As previously mentioned, there was no significant change in the fluorescence intensity of the NAYA compound during cooling, while the intrinsic fluorescence intensity of the Syn protein varied and slightly increased (Fig. 3C and D). By examining data under steady-state conditions for the Syn protein and noting the decrease in Rayleigh scattering values up to 45 ºC (Fig. 1B), it is evident that the significant drop in Syn protein aggregation when cooling is caused by a higher level of disaggregation. Hence, the outcomes achieved through fast-mixing temperature-jumps correspond to those obtained under steady-state conditions.

**Fig. 4:**
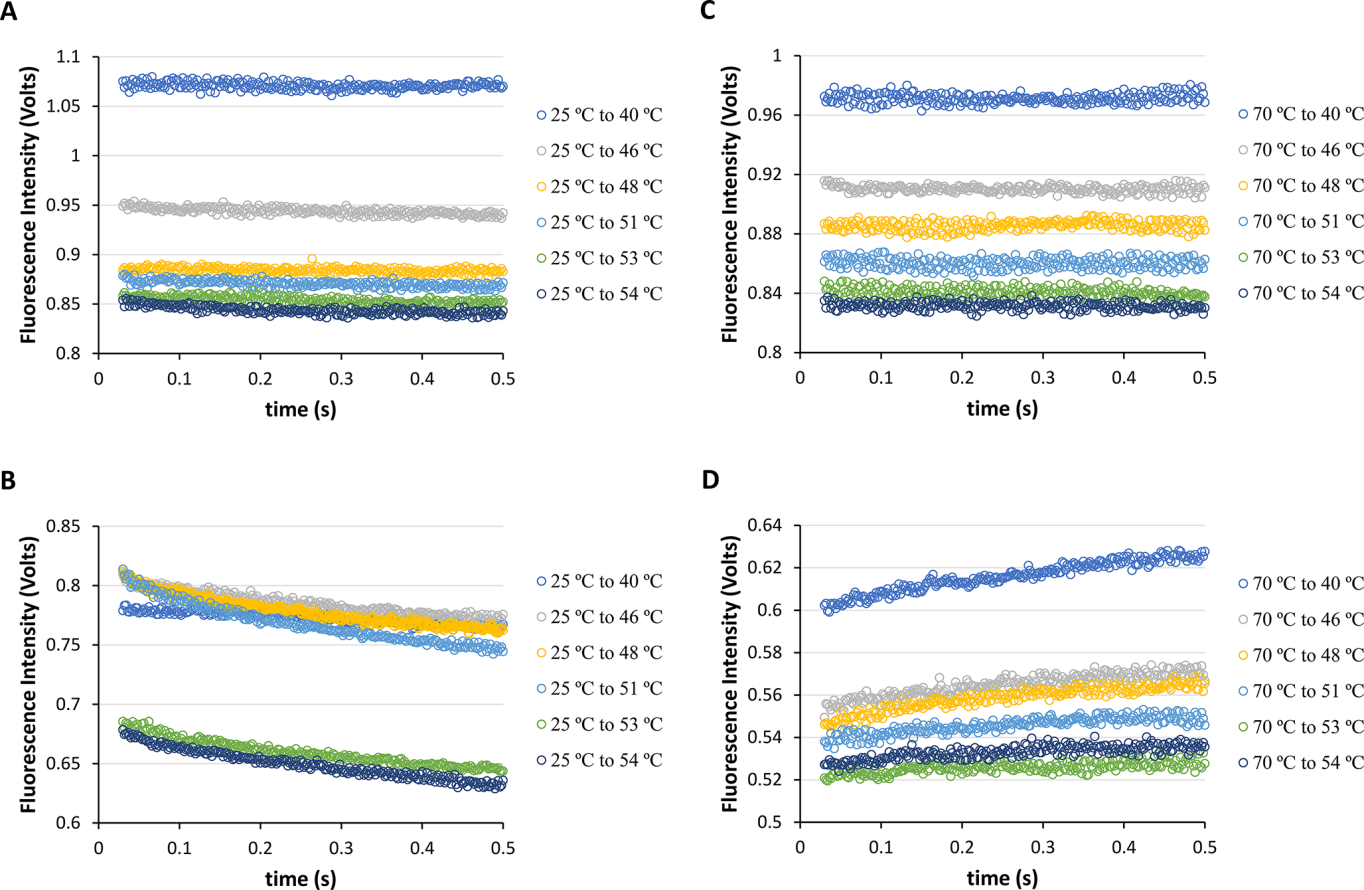
Fast-mixing temperature-jumps for the NAYA parent compound and for the Syn protein (10 mM tris-HCl, pH 7; *A*_275 nm_ = 0.4) in the first 30–500 milliseconds. (**A**) NAYA fluorescence intensity variation in the temperature-jumps from *T*_i_ = 25 ºC to *T*_f_ = 40, 46, 48, 51, 53 and 54 ºC (heating). (**B**) Syn intrinsic fluorescence intensity variation in the temperature-jumps from *T*_i_ = 25 ºC to *T*_f_ = 40, 46, 48, 51, 53 and 54 ºC (heating). (**C**) NAYA fluorescence intensity variation in the temperature-jumps from *T*_i_ = 70 ºC to *T*_f_ = 40, 46, 48, 51, 53 and 54 ºC (cooling). (**D**) Syn intrinsic fluorescence intensity variation in the temperature-jumps from *T*_i_ = 70 ºC to *T*_f_ = 40, 46, 48, 51, 53 and 54 ºC (cooling). The temperature-jumps were performed in triplicate and for clarity only the average of the results is presented

Then, it was decided to use a higher starting temperature (*T*_i_) of 25 ºC and based on our previous research on equilibrium, it was found that Syn protein disaggregation occurred initially under room temperature conditions [6]. Our plan is to rapidly change the temperature from 25 ºC using fast-mixing temperature-jumps to see if Syn protein disaggregation continues to occur. For this goal, it was conducted fast-mixing temperature-jumps from *T*_i_ = 25 ºC to *T*_f_ = 40, 46, 48, 51, 53, and 54 ºC (heating) for both NAYA compound and Syn buffered solutions at pH 7 (Fig. 4A and B). Likewise, in relation to Fig. 3A, in Fig. 4A there was no significant variation in the recorded NAYA fluorescence intensity during the first 500 milliseconds when heating. Unlike the previous figures, Fig. 4B shows a reduction in the intrinsic fluorescence intensity recorded over time. This outcome shows that Syn protein disaggregation is occurring within the initial 500 milliseconds of the temperature-jumps being carried out. In the end, it was opted for a 70 ºC initial temperature and proceeded to cool the Syn protein solutions to see its impact on amyloid protein aggregation, in particular. Hence, it was conducted fast-mixing temperature-jumps in NAYA compound and Syn buffered solutions at pH 7, ranging from initial temperature (*T*_i_) of 70 ºC to final temperatures (_f_) of 40, 46, 48, 51, 53 and 54 ºC (cooling) (Fig. 4C and D). Similarly, to Fig. 3D, in Fig. 4D there is a rise in the Syn intrinsic fluorescence intensity observed over time during the cooling process. This outcome suggests that Syn aggregation happens within the first 500 milliseconds of the temperature-jumps when cooling. Taking into account that Syn protein disaggregation happens during heating and protein aggregation is seen during cooling, it can be concluded that there might be an equal importance placed on the Syn protein aggregation and disaggregation events at the higher temperatures studied. This aligns with the findings under steady-state conditions where there was some minor protein aggregation seen at temperatures exceeding 45 ºC (Fig. 1B).

**Fig. 5:**
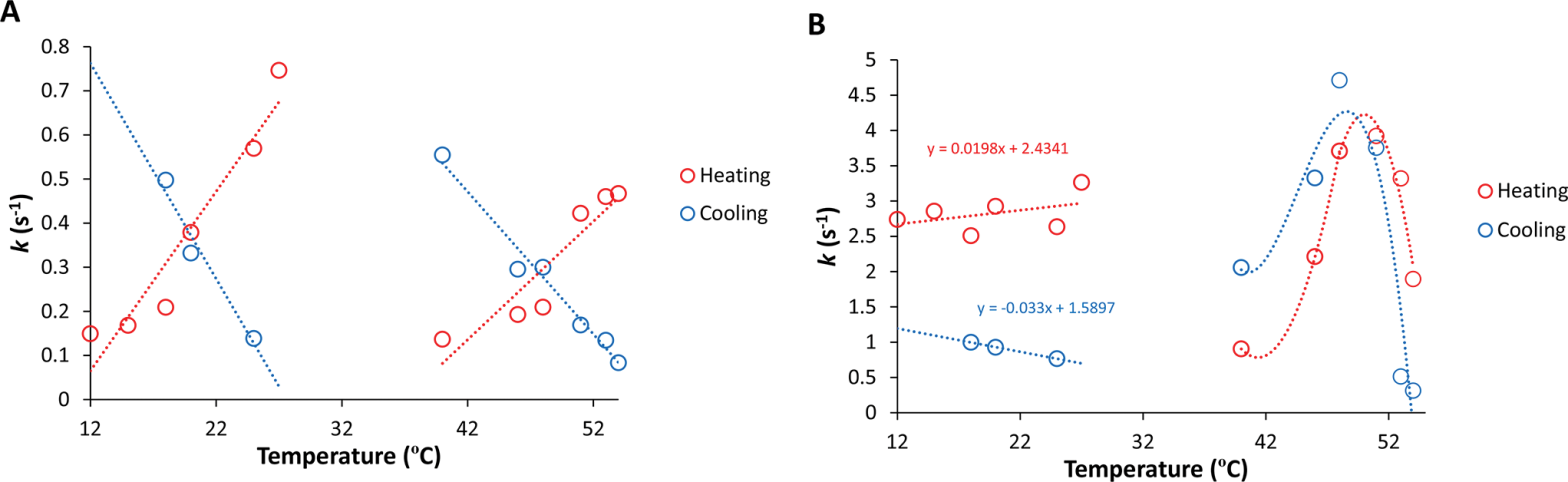
Calculated first-order rate constants for the NAYA compound (**A**) and Syn protein (**B**) from the fluorescence intensity data depicted in Figs. 3 and 4 (heating and cooling processes are indicated)

In the short time scale of 30–500 milliseconds, it was observed that lower temperatures encourage the aggregation of Syn, while higher temperatures promote its disaggregation (Figs. 3 and 4). For small molecules, the kinetic process is preferred at lower temperatures while the thermodynamic process (solution entropy) is favored at higher temperatures [35]. This situation appears to also be relevant to the Syn protein as well. Additionally, it was determined the first-order rate constants for the fluorescence intensity data shown in Figs. 3 and 4. For both the NAYA parent compound and the Syn protein, the analysis was conducted during the first 500 milliseconds (Fig. 5). Figure 5A displays the first-order rate constants for the NAYA compound in heating and cooling processes, indicating an increase in these small rate constants in both processes. The rise in the calculated first-order rate constants is slightly greater at lower temperatures compared to higher temperatures (Fig. 5A). Figure 5B shows the calculated first-order rate constants for the Syn protein during the heating and cooling processes. In contrast to the NAYA compound (Fig. 5A), the calculated first-order rate constants show different trends at low and high temperatures (Fig. 5B). At low temperatures, the first-order rate constants increase when cooling compared to heating, within experimental error (Fig. 5B). During higher temperatures observed, the calculated first-order rate constants seem to follow a comparable pattern in both the heating and cooling processes, with an initial increase followed by a decline in the rate constants (Fig. 5B). A detailed observation shows that the change in the determined first-order rate constants is symmetrical in heating and cooling processes (Fig. 5B). Additionally, in the Syn system, at lower temperatures, the main equilibrium is between the NAYA-like intramolecularly hydrogen-bonded arrangement in β-sheets (Scheme 1C) and its disrupted or extended form (Scheme 1B). In this scenario, understanding the protein system is quite straightforward with minimal impacts (kinetics) influencing the processes of protein aggregation and disaggregation. In the elevated temperature range, there are indeed discontinuous lines for the calculated first-order rate constants. This suggests that Syn protein aggregation and disaggregation involve more effects, such as kinetic effects, solvent effects due to decreased dielectric constant, and thermodynamic effects due to increased solution entropy with rising temperature. Even with these effects, the discontinuous lines representing the determined first-order rate constants appear quite alike, suggesting that protein aggregation and disaggregation have similar characteristics. Ultimately, some of the effects mentioned may offset each other when heating and cooling at higher temperatures.

### Fast-Mixing Temperature-Jumps for the Syn Protein in the Burst Phase Under Stopped-Flow Spectrometry Conditions

**Fig. 6:**
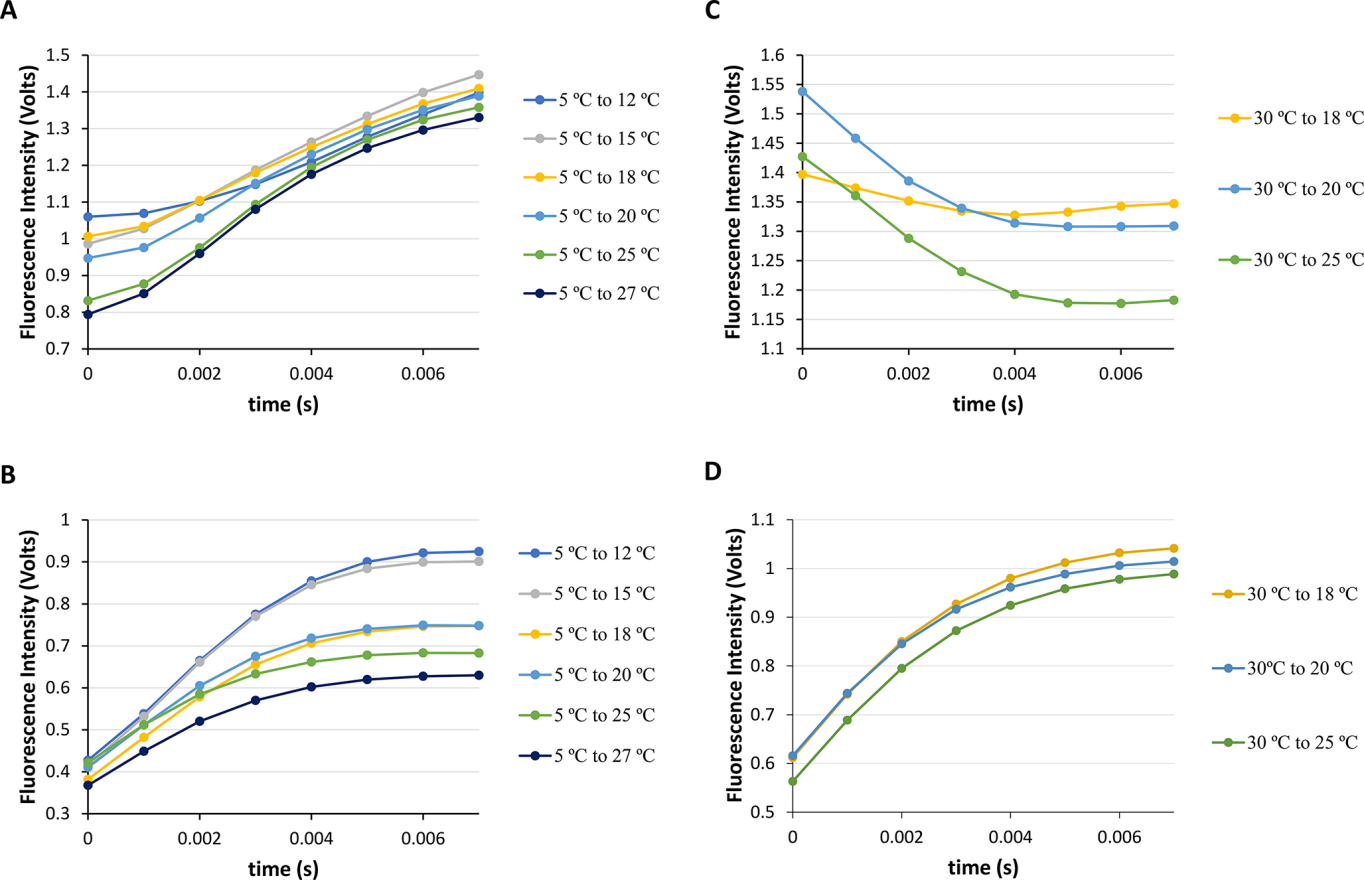
Fast-mixing temperature-jumps for the NAYA parent compound and for the Syn protein (10 mM tris-HCl, pH 7; *A*_275 nm_ = 0.4) in the first 7 milliseconds of the burst phase. (**A**) NAYA fluorescence intensity variation in the temperature-jumps from *T*_i_ = 5 ºC to *T*_f_ = 12, 15, 18, 20, 25 and 27 ºC (heating). (**B**) Syn intrinsic fluorescence intensity variation in the temperature-jumps from *T*_i_ = 5 ºC to *T*_f_ = 12, 15, 18, 20, 25 and 27 ºC (heating). (**C**) NAYA fluorescence intensity variation in the temperature-jumps from *T*_i_ = 30 ºC to *T*_f_ = 18, 20 and 25 ºC (cooling). (**D**) Syn intrinsic fluorescence intensity variation in the temperature-jumps from *T*_i_ = 30 ºC to *T*_f_ = 18, 20 and 25 ºC (cooling). The temperature-jumps were performed in triplicate and for clarity only the average of the results is presented

During the quiescent phase (ranging from 30 to 500 milliseconds), temperature-jumps in fast-mixing experiments conducted in stopped-flow spectrometry revealed that the Syn protein forms aggregates at lower temperatures, and disaggregates at higher temperatures, as observed from changes in intrinsic fluorescence intensity (Figs. 3 and 4). In this section, it will be examined the events happening during the burst phase (up to approximately 10 milliseconds), utilizing the identical approach. As it was stated during the quiescent phase, it was again conducted fast-mixing temperature increases in buffered NAYA compound and Syn protein solutions at pH 7, raising the temperature from *T*_i_ = 5 ºC to *T*_f_ = 12, 15, 18, 20, 25 and 27 ºC (heating) (Fig. 6A and B). In Fig. 6A, an increase in intrinsic fluorescence intensity is observed within the first 7 milliseconds, followed by a continuous rise until reaching 20 milliseconds during the burst phase. It was previously deduced that the events in the burst stage are reminiscent of the initial 20 milliseconds, after which the quiescent phase can commence [2]. And, these conditions are seen in the NAYA parent compound but not in the Syn protein, as we will see later on. It was recently reported that the tyrosyl group rotamers conformational model is useful in explaining the events in the burst phase [2]. Unlike in the aqueous solutions, where all three tyrosyl group rotamers coexist simultaneously [34], in an active phase like the burst phase, they can be isolated temporally [2]. Specifically, the *gauche*(+) rotamer, known to be the less stable rotamer with the tyrosyl group more exposed to water molecules, is associated with the initial and lowest NAYA compound fluorescence intensity within around 2 milliseconds (Fig. 6A). The *trans* and *gauche*(−) tyrosyl group rotamers have a longer lifespan as they are not as exposed to solvent molecules like water, leading to a higher fluorescence intensity of the NAYA compound after 2 milliseconds (Fig. 6A). According to Fig. 6A, the NAYA tyrosyl group detects a subtle presence of water during the initial heating process at lower temperatures. In Fig. 6B, there is a noticeable rapid increase in Syn intrinsic fluorescence intensity within the initial 7 milliseconds, which differs from the behavior of the NAYA compound under similar conditions. So, primarily the Syn tyrosyl groups rotamers *trans* and *gauche*(−), which have a longer lifespan, remain in the turbulent period during the initial 7 milliseconds of the burst phase. This suggests that the Syn protein tyrosyl groups are exposed to a hydrophobic environment that is more pronounced than what is seen for the NAYA compound tyrosyl group. This behavior is expected because the NAYA compound’s intramolecular hydrogen bonding configuration detects a more water-like environment compared to the intramolecular hydrogen bonding interactions similar to NAYA present in the hydrophilic face of β-sheets within the Syn amyloid precursor forms structures. It is worth noting that the burst phase turbulence regime for lower temperatures is observed within the first 7 milliseconds, as the fluorescence intensity of the Syn protein decreases after this time period. It is unclear why this event happens during the burst phase specifically for Syn and not for the NAYA compound. We expect that the drop in Syn fluorescence intensity after 7 milliseconds during the burst phase may be linked to higher viscosity in the protein solutions. Moreover, it was performed fast-mixing cooling experiments in buffered NAYA compound and Syn protein solutions at pH 7, decreasing the temperature from *T*_i_ = 30 ºC to *T*_f_ = 18, 20, and 25 ºC (cooling) (Fig. 6C and D). Figure 6C illustrates the reduction in fluorescence intensity of the NAYA compound within the initial 7 milliseconds. The decrease in fluorescence intensity is mostly associated with the increased population of the *gauche*(+) rotamer, which has a lifespan related to the early turbulent period in the burst phase when tyrosyl groups are likely to be completely exposed to water molecules. Hence, in Fig. 6C, the decrease in fluorescence intensity of the NAYA compound suggests that the tyrosyl group is completely exposed to water molecules. This aspect makes sense because by heating the solutions containing the NAYA compound, the intramolecular hydrogen-bonded structure of NAYA is disturbed, leading to a higher proportion of NAYA in a non-complexed or extended form. The latter is accountable for promoting self-association of NAYA while raising the temperature of the compound solutions. Cooling the NAYA solutions results in the NAYA system returning to its original state by decreasing NAYA self-association and forming the mentioned NAYA intramolecularly hydrogen-bonded configuration. In simpler terms, the NAYA system can be reversed by changing the temperature, which confirms the accuracy of the rate constants found for the NAYA compound at low temperatures (Fig. 5A). Similarly, to Fig. 6B and D also indicates an increase in the Syn intrinsic fluorescence intensity during the first 7 milliseconds. This implies that the system shows comparable behavior during both heating and cooling, showing that the populations of Syn tyrosyl groups *trans* and *gauche*(−) rotamers are prominent in the first 7 milliseconds, pointing to a significant hydrophobic interaction by the Syn tyrosyl groups. This is due to the absence of any disturbance in the NAYA-like hydrogen bonds within the hydrophilic face of the formed β-sheets in the Syn amyloid precursor forms. Understanding this result is difficult unless considering that the burst phase molecular processes are primarily started from the same species, i.e., the Syn protein monomers. To achieve Syn aggregation at lower temperatures with Syn protein monomers, the heating and cooling processes need to be closely related.

**Fig. 7:**
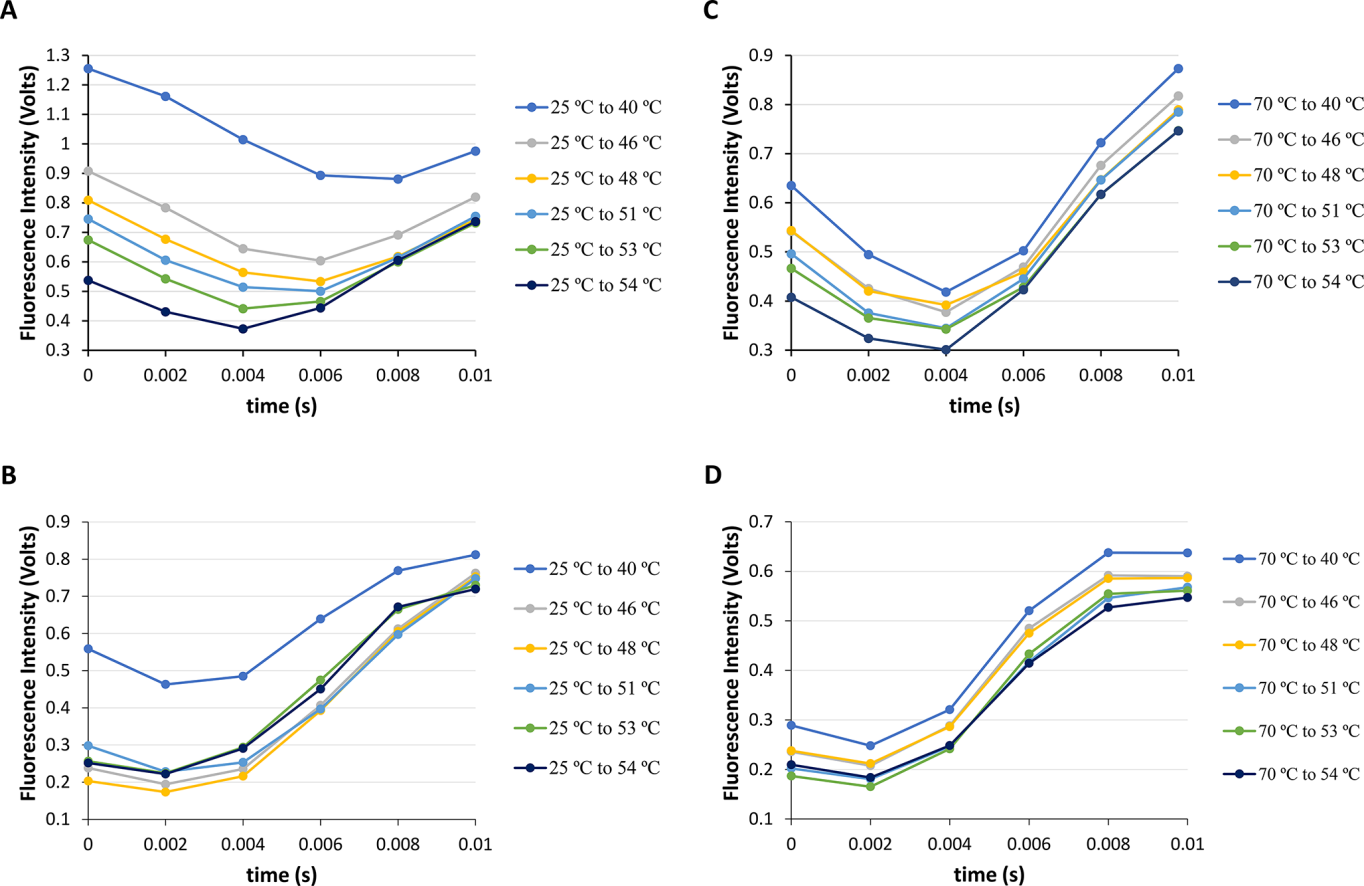
Fast-mixing temperature-jumps for the NAYA parent compound and for the Syn protein (10 mM tris-HCl, pH 7; *A*_275 nm_ = 0.4) in the first 10 milliseconds of the burst phase. (**A**) NAYA fluorescence intensity variation in the temperature-jumps from *T*_i_ = 25 ºC to *T*_f_ = 40, 46, 48, 51, 53 and 54 ºC (heating). (**B**) Syn intrinsic fluorescence intensity variation in the temperature-jumps from *T*_i_ = 25 ºC to *T*_f_ = 40, 46, 48, 51, 53 and 54 ºC (heating). (**C**) NAYA fluorescence intensity variation in the temperature-jumps from *T*_i_ = 70 ºC to *T*_f_ = 40, 46, 48, 51, 53 and 54 ºC (cooling). (**D**) Syn intrinsic fluorescence intensity variation in the temperature-jumps from *T*_i_ = 70 ºC to *T*_f_ = 40, 46, 48, 51, 53 and 54 ºC (cooling). The temperature-jumps were performed in triplicate and for clarity only the average of the results is presented

Similarly, it was conducted fast-mixing temperature increases for the NAYA compound and Syn protein buffered solutions at pH 7 from initial temperature of 25 ºC to final temperatures of 40, 46, 48, 51, 53 and 54 ºC (heating) (Fig. 7A and B). In Fig. 7a, it is evident that the presence of the NAYA tyrosyl group *gauche*(+) rotamer population, initially seen at the start of the burst phase, continues for 4–6 milliseconds. This suggests that the NAYA tyrosyl group is detecting an increased environment to water at higher temperatures compared to the one expected at lower temperatures when heated (Figs. 6A and 7A). In addition, the expected rise in solution entropy at elevated temperatures opposes the self-association of NAYA compound which could cause the NAYA tyrosyl group to perceive a more water-like environment during the burst phase in those circumstances. This implies that at higher temperatures, there are more NAYA compound intramolecularly hydrogen-bonded structures formed, causing the NAYA tyrosyl group to perceive a more water-like environment compared to lower temperatures. Figure 7B displays the change in Syn protein fluorescence intensity during the initial 10 milliseconds after heating the protein solutions in the burst phase. Similarly, to the NAYA compound that was analyzed in Fig. 7A, the Syn protein also shows a stable initial fluorescence intensity in the first 2–4 milliseconds (Fig. 7B). This demonstrates that the population of Syn protein tyrosyl groups in the *gauche*(+) rotamer conformation increases over time, leading to an exposure of the Syn tyrosyl groups to water molecules. In a similar manner to the NAYA compound, the higher temperature range results in a higher entropy of the Syn protein solutions and leads to a decrease in Syn aggregation, as shown in Fig. 1B analysis; there is only minor Syn protein aggregation at temperatures above 45 ºC. It is crucial to mention that during the burst phase, the Syn protein monomers are the main species involved in reactions, as mentioned before. In the end, it was performed fast-mixing temperature decreases in both NAYA compound and Syn buffered solutions with pH 7 from *T*_*i*_ = 70 ºC to *T*_*f*_ = 40, 46, 48, 51, 53, and 54 ºC within the initial 10 milliseconds (Fig. 7C and D). Figure 7C suggests that the NAYA tyrosyl group prefers the *gauche*(+) rotamer within the first 2–4 milliseconds, showing a lower interaction with water molecules when cooling compared to the results in Fig. 7A during heating at higher temperatures. Referring to the cooling process in Fig. 7C indicates a reduction in the solution entropy, leading to increased self-interactions among NAYA compound molecules. In Fig. 7D, it is displayed how the fluorescence intensity of Syn tyrosyl groups changes within the initial 10 milliseconds of the burst phase while cooling at higher temperatures. The change in fluorescence intensity of Syn tyrosyl groups is actually comparable to the variation shown in Fig. 7B during heating at higher temperatures. Similar behavior is observed in the burst phase of heating and cooling for the Syn protein, which is primarily linked to the reactivity of Syn monomer species, as previously seen at lower temperatures (Fig. 6B and D). It was previously mentioned that the Syn protein fluorescence intensity decreases during the burst phase at lower temperatures for over 7 milliseconds (Fig. 6B and D). This was because the protein solution viscosity could potentially increase as a result of the turbulence during the burst phase. The forecast proves accurate as the recorded fluorescence intensity of Syn protein rises with higher temperatures, peaking at 10 milliseconds (Fig. 7B and D). Because the temperature affects the viscosity of the protein solution, our hypothesis is confirmed: the increase in Syn protein fluorescence intensity lasts longer in the burst phase at higher temperatures compared to lower temperatures.

**Fig. 8:**
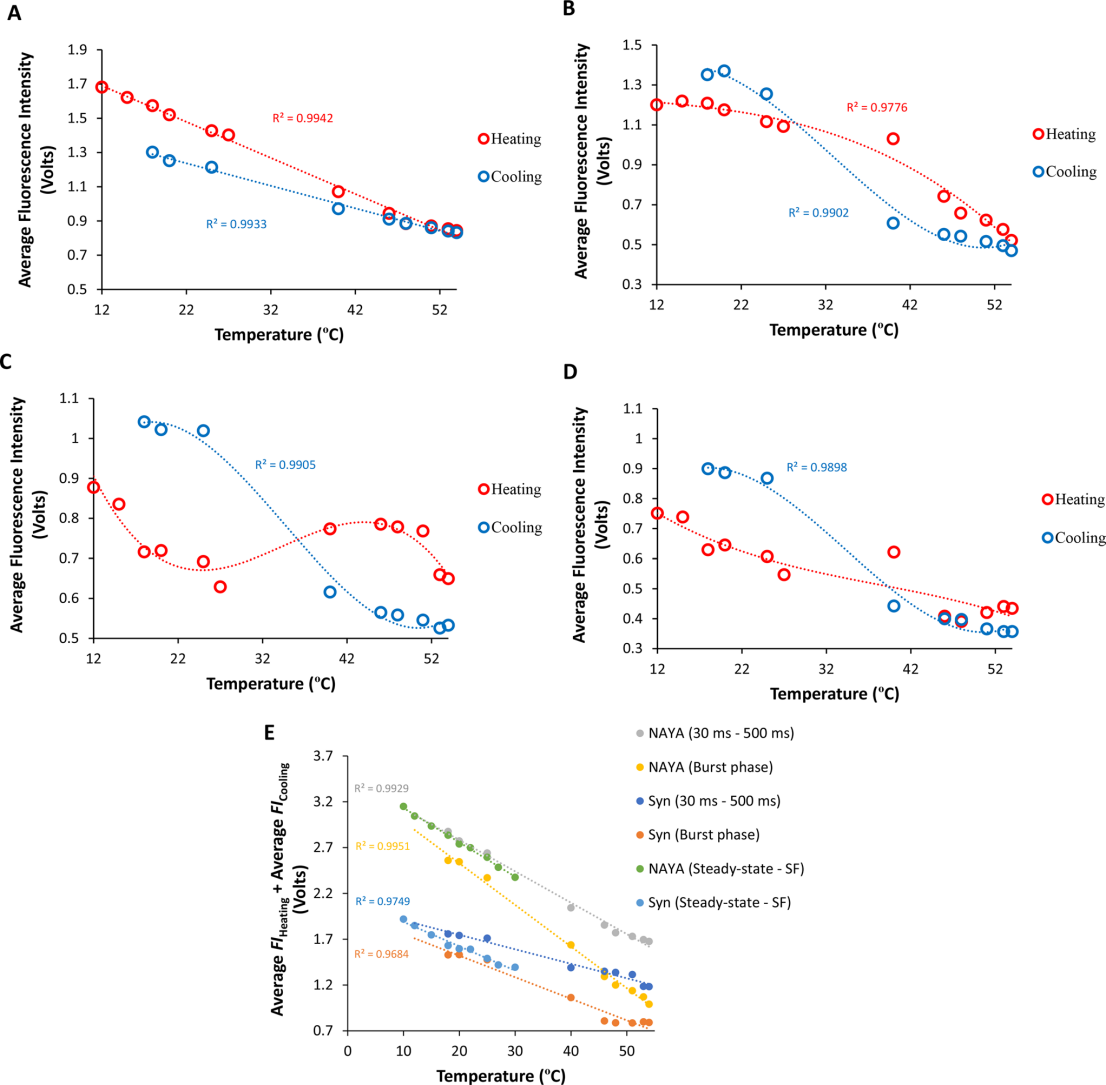
Calculated average fluorescence intensity values for both the NAYA parent compound and the Syn protein from data depicted in Figs. 3, 4, 6 and 7 as function of the final temperature of the performed fast-mixing temperature-jumps. (**A**) NAYA compound in the quiescent phase (from 30 to 500 milliseconds) during heating and cooling. (**B**) NAYA compound in the burst phase (up to 10 milliseconds) during heating and cooling. (**C**) Syn protein in the quiescent phase (from 30 to 500 milliseconds) during heating and cooling. (**D**) Syn protein in the burst phase (up to 10 milliseconds) during heating and cooling. (**E**) Combined average fluorescence intensity (FI) values for the NAYA compound and the Syn protein from data in (**A**), (**B**), (**C**) and (**D**). Steady-state fluorescence intensity data obtained by the stopped-flow (SF) method for the NAYA compound and for the Syn protein in the temperature range of 10 ºC to 30 ºC are also presented

Furthermore, it was calculated the average of the fluorescence intensity data shown in the Figs. 3, 4 and 6, and 7. In Fig. 8, it is present the final temperatures measured during the fast-mixing temperature-jumps and the calculated average fluorescence intensity values for both the NAYA parent compound and the Syn protein. Figure 8A shows that, during the quiescent phase (from 30 to 500 milliseconds), the average fluorescence intensity values tend to be closer at higher temperatures and more divergent at lower temperatures. The complexity of the subject arises from the fluctuations in both the entropy of the solution and the dielectric constant of the medium, which lead to the diverse behaviors observed in Fig. 8 during heating and cooling at both low and high temperatures. To analyze the system from a different perspective, it was opted to combine the average fluorescence intensity values of the NAYA compound and the Syn protein in both the quiescent and burst phases for clarity (Fig. 8E). In Fig. 8E, it is evident that the combined average fluorescence intensity of the NAYA compound decreases more than the combined average fluorescence intensity of the Syn protein as the fast-mixing temperature-jumps reach their final temperature. Similarly, to steady-state data shown in Fig. 1, it was also measured the fluorescence intensity of the NAYA parent compound and the Syn protein as the solutions were heated using stopped-flow spectrometry. More precisely, without making fast-mixing temperature-jumps, it was measured the fluorescence intensity by stopped-flow spectrometry and conducted experiments at temperatures between 10 ºC and 30 ºC (Fig. 8E). It was found that the fluorescence intensity measured in the stopped-flow and the average fluorescence intensity values during the quiescent phase for the NAYA compound were comparable (Fig. 8E). In contrast, there was a discrepancy between the steady-state fluorescence intensity observed in the stopped-flow method and the average fluorescence intensity values during the quiescent phase for the Syn protein (Fig. 8E). In this scenario, as the temperature recorded increased, the data from steady-state and burst phases obtained through fast-mixing temperature-jumps became more similar (Fig. 8E). This indicates that in the steady-state, the reactivity of the Syn monomeric protein is crucial; therefore, it was previously determined that reactions originate from the Syn monomeric protein during the burst phase.

## Conclusions

This report utilized rapid temperature changes within the initial 500 milliseconds by stopped-flow spectrometry to study the early aggregation of the small amyloid Syn protein in neutral pH buffered solutions. For this aim, it was also contrasted the findings for the Syn protein with those achieved for the NAYA parent compound in the same circumstances. In this study, a molecular mechanism basis was identified for the NAYA compound system and the Syn protein system. This molecular mechanism basis is comprised of the existence of NAYA compound intramolecular hydrogen-bonded structures and NAYA-like intramolecular hydrogen-bonded interactions in the hydrophilic face of β-sheets. The latter structures can be found in the Syn amyloid precursor forms structures during the early stages of the Syn protein aggregation. Moreover, the rapid temperature changes examined by stopped-flow spectrometry were compared to steady-state results using a spectrofluorimeter. The rapid changes in temperature during heating and cooling periods, lasting between 30 and 500 milliseconds in the quiescent phase, lead to the formation and breakage of Syn aggregates. First-order rate constants calculated in still conditions show that Syn aggregation and disaggregation processes are linked and play a role in the formation of minor Syn aggregates at the higher temperatures studied. Regarding the burst phase (up to 10 milliseconds), the results were more difficult to interpret, and it was determined that the reactivity of Syn monomers primarily causes Syn protein aggregation. We also stress that the burst phase can provide insights into how this protein aggregates in solution on a very short timescale. The burst phase can be crucial for examining the processes in amyloid proteins aggregation, and additional detection methods like light scattering, circular dichroism, and conductivity measurements in stopped-flow spectrometry can provide further insights into these complex systems.

## Electronic Supplementary Material

Below is the link to the electronic supplementary material.

Supplementary Material 1

## Data Availability

No datasets were generated or analysed during the current study.
