# Supplementary material for: Temperature-Driven Stopped-Flow Experiments for Investigating the Initial Aggregation of the α-Synuclein Amyloid Protein, Focusing on Active and Inactive Phases
Source: J Fluoresc. 2024 Oct 2;35(7):5817–32. doi: 10.1007/s10895-024-03971-8 (PMC12325386; doi:10.1007/s10895-024-03971-8)
Supplement: Supplementary file 1 — Supplementary Material 1 [file 10895_2024_3971_MOESM1_ESM.docx]

**Supplementary Material**

Temperature-driven stopped-flow experiments for investigating the initial aggregation of the α-synuclein amyloid protein, focusing on active and inactive phases

Marco A. Saraiva ^1,2 *^

^1^ Centro de Química Estrutural, Institute of Molecular Sciences, Instituto Superior Técnico, University of Lisbon, 1049-001 Lisbon, Portugal

^2^ Instituto de Tecnologia Química e Biológica António Xavier, Universidade Nova de Lisboa, Av. da República, 2780-157 Oeiras, Portugal

* Correspondence e-mail: [marco.saraiva@tecnico.ulisboa.pt](mailto:marco.saraiva@tecnico.ulisboa.pt)

**Supplementary text, Figure S1 and Table S1.**

In 2020, we initially noted the development of early sizable Syn aggregates, possibly Syn amyloid precursor forms, in the protein solutions within less than 1 hour through DLS (with a hydrodynamic radius exceeding 100 nm) [1]. The Syn protein stock solutions were centrifuged using 100 kDa membrane centrifuge filters, thus protein aggregates are detected early in the solution, not from potential impurities in the purification process [1]. It should be emphasized that early-stage aggregates of Syn are responsive to changes in pH and ionic strength in protein solutions [1–3].

However, it was performed an additional DLS experiment in this study once it had been centrifuged the Syn protein stock solutions with 100 kDa membrane centrifuge filters. It was once again noticed the presence of early Syn aggregates, as demonstrated in the intensity-based size distributions (Fig. S1A). It was essential for us to incorporate the initial DLS measurement, revealing two less broad peaks representing the Syn monomer species and the Syn aggregates. Still, the exact characteristics of these sizable Syn aggregates are unknown. Fig. S1B shows the size distributions based on number, indicating that Syn monomer species dominate in protein solutions, with Syn aggregated species making up less than 0.01% of the total Syn species in solution. Therefore, by using a 33.5 µM Syn monomer concentration in the DLS experiments, the concentration of the Syn aggregates mentioned would be around 10^−9^ M to 10^−10^ M, which is four orders of magnitude lower than the concentration of protein monomers. It was chosen to investigate the DLS data in more detail, particularly the intensity autocorrelation functions shown in Figure S1C, and it was observed that the initial DLS measurement did not have a fit distribution that was accurate according to the DLS software. The Malvern general purpose algorithm was utilized with an α parameter of 0.01 as the “regularizer” and a quadratic weighting scheme. Therefore, the specified α parameter value is most appropriate for examining protein samples with Malvern instruments, as demonstrated with lysozyme (0.3 mg/mL in PBS buffer at pH 6.8) and denatured hemoglobin at 44 ºC (PBS buffer at pH 6.8). Because the algorithm utilizes a quadratic weighting system, it was chosen to model the autocorrelation function for the initial DLS measurement by incorporating three exponentials in the simulation. Hence, the mathematical rationalization is explained as follows.

DLS quantifies fluctuations in light intensity over time by measuring a second-order correlation function, *g*^(2)^ (τ). The intensity function is adjusted by a delay time (τ) and then the autocorrelation function g(τ) is computed. The correlation function mentioned for a monodisperse sample can be expressed through the following equation:

$g^{\left( 2 \right)}\left( \tau\right)=1+\beta exp(-2\Gamma\tau)$ (1)

Where *β* is the correlation function at zero delay, Γ is the correlation function decay rate, and the baseline of the correlation function relaxes to a value of 1 at infinite delay. The correlation function decay rate, Γ, can be converted to the translational diffusion coefficient, *D*_t_, for the particle through the relation:

$D_{t}=\frac{\Gamma}{q^{2}}$ (2)

Thus, *q* is the magnitude of the scattering vector, and is given by:

$\left| q \right|=\frac{4\pi n_{0}}{\lambda_{0}} \sin\left( \frac{\theta}{2} \right)$ (3)

where *n*_0_ is the solvent index of refraction, λ_0_ is the vacuum wavelength of the incident light, and θ is the scattering angle. Also, the diffusion coefficient, *D*_t_, can be interpreted as the hydrodynamic radius, *R*_h_, of a diffusing sphere via the Stokes-Einstein equation:

$R_{h}= \frac{k_{B}T}{6\pi\eta D_{t}}$ (4)

Where *k*_B_ is the Boltzmann constant (1.381 × 10^‒23^ J K^‒1^), *T* is the temperature in Kelvin, and η is the absolute (or dynamic) viscosity of the solvent.

As mentioned above, the second-order correlation function *g*^(2)^(τ) can be related to the autocorrelation function *g*(τ) through the expression:

$g^{\left( 2 \right)}\left( \tau\right)-1=g\left( \tau\right)=\beta\exp(-2Г\tau)$ (5)

The aforementioned explanation is applicable to a monodisperse sample examined using DLS. However, in the case of polydisperse samples like the Syn solutions analyzed by DLS, the autocorrelation function is the result of multiple exponential decays within it. Hence, considering the presence of three exponentials in the autocorrelation functions in the current system, equation 6 can be adjusted as follows:

$g (\tau)=\beta\exp\left( -2\Gamma_{1}\tau\right)+\beta\exp\left( -2\Gamma_{2}\tau\right)+\beta\exp\left( -2\Gamma_{3}\tau\right)$ (6)

In addition, due to the definition of *β* it was further normalized all correlation functions obtained to unit and, therefore, equation 6 is now given by:

$g\left( \tau\right)=\frac{\left( 1\times\exp\left( -2\Gamma_{1}\tau\right) \times a_{1}+1\times\exp\left( -2\Gamma_{2}\tau\right)\times a_{2} +1\times\exp\left( -2\Gamma_{3}\tau\right)\times a_{3} \right)}{a_{1}+a_{2}+a_{3}}$ (7)

Therefore, equation 7 allows for the simulation of autocorrelation functions seen in Syn solutions with DLS conditions, enabling the extraction of decay rates (Γ_1_, Γ_2_, and Γ_3_) and exponential coefficients (*a*_1_, *a*_2_, and *a*_3_) (Table S1). In Fig. S1D, it is shown the normalized autocorrelation function obtained for the initial DLS measurement and corresponding fit distribution. From Table S1, the hydrodynamic diameter *d_h_* (*d_h_* = 2*R_h_*) of the Syn particles in solution in the simulated normalized autocorrelation function was determined from the calculated decay rates, as mentioned above, and these decay rates were obtained through interpolation for single scatterers.

In fact, the hydrodynamic diameters calculated for the heavier Syn particles in solution (Table S1), denoted as *d_h1_*, *d_h2_*, and *d_h3_*, suggested the movement of a lone scatterer in DLS circumstances. Accordingly, to the values of *d_h3_* (710 nm) and *d_h2_* (3.3 nm), this one scatterer might be an elongated Syn species present in the solution (Table S1). By this explanation, we suggest that the elongated Syn species referred to could possibly be amyloid precursor forms found in the Syn solutions. In literature, when *qL* is much greater than 1, seen in diluted solutions of thin rods where the length is similar to wavelength, *D*t = Γ / *q*^2^ → *D*_┴_ + (*L*^2^ / 12)*D*r [4]. This indicates the relationship between *D*_‖_, *D*_┴_, and *D*r with the length *L* and width *d* of the rod [4]. This means that translational diffusion *D*t is linked to rotational diffusion *D*r. As a result, for thin rods with a length similar to the wavelength, all diffusion coefficients and their geometrical dimensions can be determined by DLS [4]. Indeed, in the simulation presented for the autocorrelation function from the initial DLS measurement, the elongated amyloid precursor form size matches the wavelength of incoming light (633 nm), as shown by measurements like *d_h3_* in Table S1. Furthermore, accurately determining the length and width (geometrical dimensions) of elongated amyloid precursor form, like in our simulation of autocorrelation functions from the initial DLS measurement, can only be achieved due to the coupling of translational diffusion *D*t with rotational diffusion *D*r in the decay rates, as mentioned.

It is important to mention that due to the very low concentration of early elongated amyloid precursor forms detected in this report (i.e., approximately 10^−9^ – 10^−10^ M) methods like X-ray diffraction and solid-state NMR are not helpful for characterizing the referred elongated amyloid precursor forms, as the signals observed are solely from the Syn monomer. This concentration is also much lower than the detection limits documented for atomic force microscopy (AFM) and transmission electron microscopy (TEM) [5,6]. Reported protein concentrations in EM grids and AFM typically range from approximately 10^−7^ to 10^−8^ M [5,6]. Therefore, the current simulation of the obtained autocorrelation function for the initial DLS measurement represents a new strategy (and yet to be further explored) to gather information of the size parameters for the early-formed elongated amyloid precursor forms.

**Figure S1.** DLS analysis of Syn protein solutions at pH 7 over time. (A) Determined size distributions based on intensity for eight DLS measurements up to 24 minutes for a 33.5 µM Syn protein concentration (*A*_275 nm_ = 0.2; ε = 5974 M^‒1^ cm^‒1^) at 20.0 ºC. (B) Determined size distributions based on number for (A). (C) Obtained autocorrelation functions for (A). (D) Normalized autocorrelation function for the initial DLS measurement and corresponding simulations (fit) due to the application of the equation 7.

**Table S1.** Normalized exponential coefficients (*a*_1_, *a*_2_ and *a*_3_), decay rates (Γ_1_, Γ_2_ and Γ_3_) and determined hydrodynamic diameters (*d_h_*_1_, *d_h_*_2_ and *d_h_*_3_) for the heavier Syn particles in solution by the application of equation 7 to the normalized autocorrelation function (Fig. S1D) for the Syn solution at pH 7. The χ^2^ value is also indicated.

| **pH** | ***a_1_*** | ***a_2_*** | ***a_3_*** | **Γ_1_** | **Γ_2_** | **Γ_3_** | ***d_h1_* (nm)** | ***d_h2_* (nm)** | ***d_h3_* (nm)** | **χ^2^** |
| --- | --- | --- | --- | --- | --- | --- | --- | --- | --- | --- |
| **7.0 (2.83 min)** | 0.12 | 0.43 | 0.45 | 15985 | 881.32 | 4.1530 | 0.18 | 3.3 | 710 | 0.12 |

**References**

1. M.A. Saraiva, Interpretation of α-synuclein UV absorption spectra in the peptide bond and the aromatic regions, *J. Photochem. Photobiol. B* 212 (2020), 112022.

2. M.A. Saraiva, Evidence of the existence of micellar-like aggregates for α-synuclein, *Int. J. Biol. Macromol.* 177 (2021) 392–400.

3. M.A. Saraiva, M.H. Florêncio, Buffering capacity is determinant for restoring early α-synuclein aggregation, *Biophys. Chem.* 282 (2022) 106760.

4. R. Alexander-Katz, Light scattering and its applications in polymer characterization, in *Handbook of polymer synthesis, characterization, and processing*, E. Saldívar-Guerra and E. Vivaldo-Lima (Eds.), John Wiley & Sons, New Jersey, 2013, pp 382.

# 5. Y.D. Ivanov, T.O. Pleshakova, I.D. Shumov, A.F. Kozlov, I.A. Ivanova, A.A. Valueva, V.Y. Tatur, M.V. Smelov, N.D. Ivanova, V.S. Ziborov, AFM imaging of protein aggregation in studying the impact of knotted electromagnetic field on a peroxidase, *Sci. Rep.* 2020, 10, 9022.

# 6. M. Rames, Y. Yu, G. Ren, Optimized negative staining: a high-throughput protocol for examining small and asymmetric protein structure by electron microscopy, *J. Vis. Exp.* 2014 (90) e51087.
